# Photoinduced Cu(II)-Mediated RDRP to P(VDF-*co*-CTFE)-*g*-PAN

**DOI:** 10.3390/polym10010068

**Published:** 2018-01-13

**Authors:** Xin Hu, Guopeng Cui, Ning Zhu, Jinglin Zhai, Kai Guo

**Affiliations:** 1 College of Materials Science and Engineering, Nanjing Tech University, Nanjing 211800, China; xinhu@njtech.edu.cn; 2 College of Biotechnology and Pharmaceutical Engineering, Nanjing Tech University, Nanjing 211800, China; 851032931@njtech.edu.cn (G.C.); ningzhu@njtech.edu.cn (N.Z.); 18852001050@njtech.edu.cn (J.Z.); 3 State Key Laboratory of Materials-Oriented Chemical Engineering, Nanjing Tech University, Nanjing 211800, China; 4 Jiangsu National Synergetic Innovation Centre for Advance Materials, Nanjing Tech University, Nanjing 211800, China

**Keywords:** fluoropolymer, photoinduced, copper-mediated RDRP, graft, PAN

## Abstract

Photoinduced Cu(II)-mediated reversible deactivation radical polymerization (RDRP) was employed to synthesize poly(vinylidene fluoride-*co*-chlorotrifluoroethylene)-*graft*-polyacrylonitrile (P(VDF-*co*-CTFE)-*g*-PAN). The concentration of copper catalyst (CuCl_2_) loading was as low as 1/64 equivalent to chlorine atom in the presence of Me_6_-Tren under UV irradiation. The light-responsive nature of graft polymerization was confirmed by “off-on” impulsive irradiation experiments. Temporal control of the polymerization process and varied graft contents were achieved via this photoinduced Cu(II)-mediated RDRP.

## 1. Introduction

Atom transfer radical polymerization (ATRP) [1,2,3,4] has been one of the most powerful reversible deactivation radical polymerization (RDRP) methodologies for the synthesis of well-defined polymers [5,6,7,8]. The original ATRP was conducted with a high concentration of Cu(I) catalyst (equivalent to alkyl halide initiator) in order to compensate for unavoidable radical termination reactions. A series of ATRP variants have been developed to reduce the catalyst loading. Activators regenerated by electron transfer (ARGET) ATRP [9], initiators for continuous activator regeneration (ICAR) ATRP [10], electrochemically-mediated ATRP (eATRP) [11], and Cu(0)-mediated RDRP [12,13,14,15] have been proposed to decrease the copper concentration below 100 ppm.

Photopolymerization has become increasingly popular thanks to its unique advantages in temporal and spatial control [16,17,18,19,20]. Photoinduced Cu(II)-mediated RDRP achieved remarkable progress in tailor-made polymer synthesis [21,22,23,24]. The combination of low-concentration CuBr_2_ and excess *tris*[2-(dimethylamino)ethyl]amine (Me6-Tren) exhibited outstanding efficiency in acrylate polymerizations [25,26]. Well-defined homopolymers, telechelic block copolymers, brush polymers, and sequence controlled polymers were obtained via photoinduced Cu(II)-mediated RDRP [27,28,29,30,31,32,33,34,35]. Iridium [36,37,38,39] and ruthenium [40] complexes were also found to be effective photocatalysts to mediate visible light-induced ATRP. Recently, organocatalyzed ATRP (OATRP) was established to generate metal-free polymers by using photoredox catalyst [41,42,43,44,45,46,47,48,49,50,51]. This young yet rapidly growing research area would attract broad interest from both academia and industry.

Poly(vinylidene fluoride-*co*-chlorotrifluoroethylene) (P(VDF-*co*-CTFE)) is one of the most used high-performance fluoropolymers with various applications [52]. Several kinds of chemical and physical modification strategies have been presented to improve the functionality and compatibility of fluoropolymers. ATRP was also employed to synthesize P(VDF-*co*-CTFE)-*g*-polystyrene (PS) and P(VDF-*co*-CTFE)-*g*-polyacrylates [53,54,55]. The dielectric and energy storage properties were enhanced by the introduction of graft chains, which could reduce the remnant polarization of the fluoropolymer [54]. This Cu(I)-mediated process required relative high catalyst loading and elevated reaction temperature to initiate the less active C–Cl bond in P(VDF-*co*-CTFE) backbone, which resulted in unexpected chain transfer reactions and dehydrochlorination reactions [56]. Cu(0)-mediated RDRP of acrylonitrile (AN) and methyl methacrylate (MMA) in the presence of P(VDF-*co*-CTFE) allowed colorless and purer P(VDF-*co*-CTFE)-*g*-PAN and P(VDF-*co*-CTFE)-*g*-PMMA copolymers for its mild reaction conditions and lower catalyst concentration [57,58,59]. Improvements were achieved by transferring the polymerization from a batch reactor to a copper tubular reactor, such as diminished inconsistent induction time, suppressed “hot spot” effect, and decreased copper and ligand concentration [60]. 

**Scheme 1:**
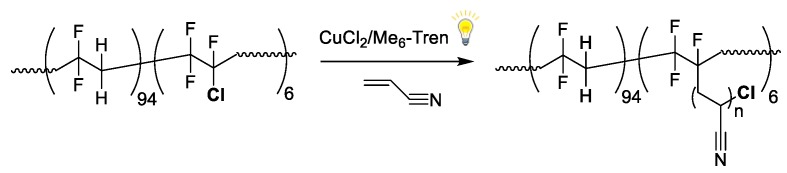
Photoinduced Cu(II)-mediated reversible deactivation radical polymerization (RDRP) to P(VDF-*co*-CTFE)-*g*-PAN. P(VDF-*co*-CTFE): poly(vinylidene fluoride-*co*-chlorotrifluoroethylene); PAN: poly(acrylonitrile).

Herein, photoinduced Cu(II)-mediated RDRP was utilized for the graft modification of P(VDF-*co*-CTFE) (Scheme 1). Polymerizations of acrylonitrile in the presence of CuCl_2_/Me_6_-Tren under UV irradiation was investigated to evaluate its effect on the preparation of P(VDF-*co*-CTFE)-*g*-PAN with low catalyst concentration and temporal control of the polymerization process.

## 2. Experimental Section

### 2.1. Materials

P(VDF-*co*-CTFE) was provided by Solvay Solex (Brussels, Belgium) (Dyneon 31008, with the [VDF]:[CTFE] = 94:6, containing 0.89 mmol chlorine atom per gram). Acrylonitrile (AN) (J&K, Beijing, China, 99%) was washed by 5 wt % aqueous sodium hydroxide solution three times, and was subsequently rinsed with deionized water until neutralization. The resultant solution was dried overnight with anhydrous MgSO_4_, then distilled under reduced pressure to remove extant inhibitor and stored under N_2_ at −20 °C. Dimethyl sulfoxide (DMSO) (Xilong Chemical, Shenzhen, China, AR) was distilled under vacuum from CaH_2_. Other reagents were used as received. The source of the UV light (Shany Cosmetics Company, New York, NY, US) setup was a commercially available UV nail gel curing lamp with four 9 W bulbs (36 W, λ_max_ ~365 nm, item model number: SH-KD-UVLAMP36W).

### 2.2. Synthesis Procedure

Polymerizations were conducted by using a Schlenk technique [57]. First, 0.500 g of P(VDF-*co*-CTFE) (VDF:CTFE = 94:6) (containing 0.445 mmol Cl atom) was dissolved in 10 mL DMSO with stirring before CuCl_2_·2H_2_O (2.4 mg, 0.0140 mmol) and Me_6_-Tren (19.2 mg, 0.0834 mmol) were added under N_2_ atmosphere. After adding 0.88 mL AN (13.35 mmol), the reactant mixture ([Cl]:[Cu]:[Me_6_-Tren]:[AN] = 1:(1/32):(6/32):30) was put into the ultraviolet light (λ_max_ ~ 365 nm) while cooling with a hair dryer. Samples were taken at regular time intervals followed by precipitation in H_2_O/CH_3_OH (*v:v* = 1:1) mixture, washed three times with ethyl alcohol, and dried overnight under reduced pressure. The resultant graft copolymer was obtained for characterization.

### 2.3. Characterization

Nuclear magnetic resonance (NMR) spectra were recorded on a Bruker (Rheinstetten, Germany) (Advance III) 400 MHz instrument for solutions in DMSO-*d*_6_ containing tetramethylsilane (TMS) as internal standard. Fourier transform infrared (FTIR) spectroscopy of polymer films was performed on a Nicolet iS5 (Thermo Scientific, Madison, WI, US). Differential scanning calorimetric (DSC) analysis was conducted on a Discovery DSC 250 (TA instruments, New Castle, DE, US). After rapid heating and cooling cycles (at a rate of 20 °C/min) to remove the thermal history, the sample was heated at a rate of 10 °C/min under nitrogen atmosphere. Thermogravimetric analysis (TGA) (TA instruments, New Castle, DE, US) results were recorded on Netzsch STA 449 F3 Jupiter (Selb, Germany) in nitrogen atmosphere at a heating rate of 10 °C/min.

## 3. Results and Discussion

Poly(acrylonitrile) (PAN) and its copolymers have been widely used as precursors for novel carbon materials with outstanding properties and performances [43]. P(VDF-*co*-CTFE)-*g*-PAN copolymers could be synthesized via Cu(0)-mediated reversible deactivation radical polymerization (RDRP) in batch and flow reactors [57,60]. Comparing with traditional ATRP protocol, copper catalyst loading was as low as about 1/4 equivalent to the chlorine atoms in the batch reactor. Copper catalyst, however, is highly desirable to decrease in order to reduce the amount of metal residue, which would potentially influence the application of final material. Inspired by the work about photoinduced Cu(II)-mediated RDRP [25,26], CuCl_2_/Me_6_-Tren was employed to promote the polymerizations of AN with P(VDF-*co*-CTFE) as macroinitiator. The polymerization results are listed in Table 1. After 6 h of UV irradiation (λ ~ 365 nm, 36 W) while cooling with a hair dryer, P(VDF-*co*-CTFE)-*g*-PAN (graft content = 20.8 mol %) was obtained with a low copper concentration ([Cl]:[Cu]:[L]:[AN] = 1:(1/32):(6/32):30) (Table 1, run 5) according to NMR analysis [57]. A 13.4 mol % graft content was achieved, even upon reducing the catalyst feed ratio into 1/64 (Table 1, run 7). This would be favorable for the applications of resultant graft copolymers in dielectric devices.

**Figure 1:**
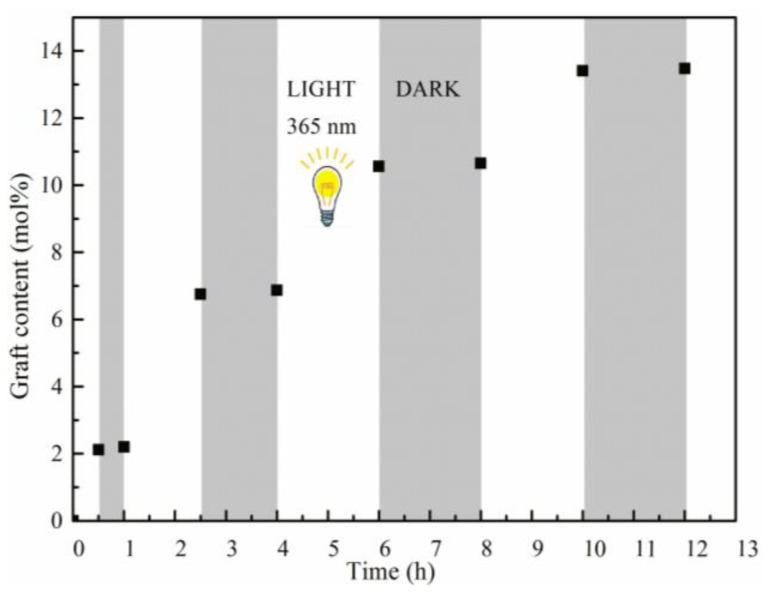
Graft contents vs. reaction time dependence for photoinduced Cu(II)-mediated RDRP of AN in DMSO with P(VDF-*co*-CTFE) as macroinitiator under “on-off” light sequence ([Cl]:[Cu]:[L]:[AN] = 1:(1/32):(6/32):30, UV (λ_max_ = 365 nm)).

Controlled experiments in the absence of light source or any reagent were conducted. P(VDF-*co*-CTFE) with no PAN graft chains was obtained by the removal of UV irradiation (Table 1, run 1). To further illustrate the light-responsive nature of the polymerization, a series of polymerizations were tested by introducing an “off-on” sequence ([Cl]:[Cu]:[L]:[AN] = 1:(1/32):(6/32):30). In Figure 1, the graft content reached 2.1 mol % after 30 min and stayed almost unchanged during 30 min dark. The re-exposure to UV enabled the polymerization to start again. These cycles were repeated several times. The omission of P(VDF-*co*-CTFE) afforded no product (Table 1, run 2), which indicated that autopolymerization of AN did not occur under the current condition. In the absence of catalyst/ligand, no graft content was observed (Table 1, run 3), which elucidated the control of activation/deactivation equilibrium by CuCl_2_/Me_6_-Tren.

**Figure 2:**
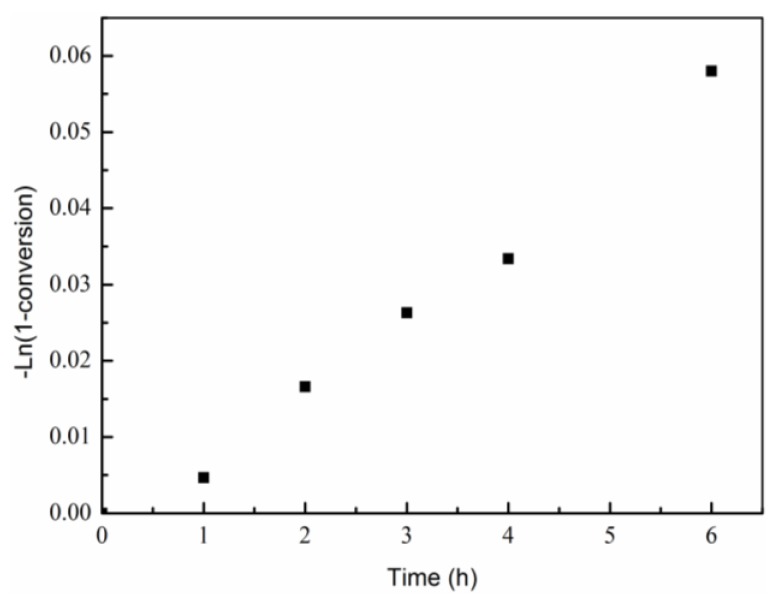
Semilogarithmic kinetic plots for photoinduced Cu(II)-mediated RDRP of AN in DMSO with P(VDF-*co*-CTFE) as macroinitiator ([Cl]:[Cu]:[L]:[AN] = 1:(1/32):(6/32):30, UV (λ_max_ = 365 nm).

The influence of [Cu]:[L] on graft polymerization was investigated. It was supposed that 1:6 would be better to yield a higher graft content (Table 1, runs 4, 5, and 6), which was consistent with previous reports [25,26]. Kinetics study showed a linear dependence between −ln(1-conversion) and reaction time. This confirmed that the polymerization rate was first-order with respect to the monomer concentration (Figure 2). Under the optimized reaction conditions, polymerizations with different monomer feed ratio were carried out to fabricate P(VDF-*co*-CTFE)-*g*-PAN with varied graft contents (Table 1, runs 8 and 9). A chain extension experiment was conducted. The resultant P(VDF-*co*-CTFE)-*g*-PAN (graft content = 20.8 mol %) was used as macroinitiator to initiate polymerization of AN ([Cl]:[Cu]:[L]:[AN] = 1:(1/32):(6/32):(30), UV (λ_max_ = 365 nm)). The graft content was increased into 30.2 mol % upon another 6 h exposure.

**Figure 3:**
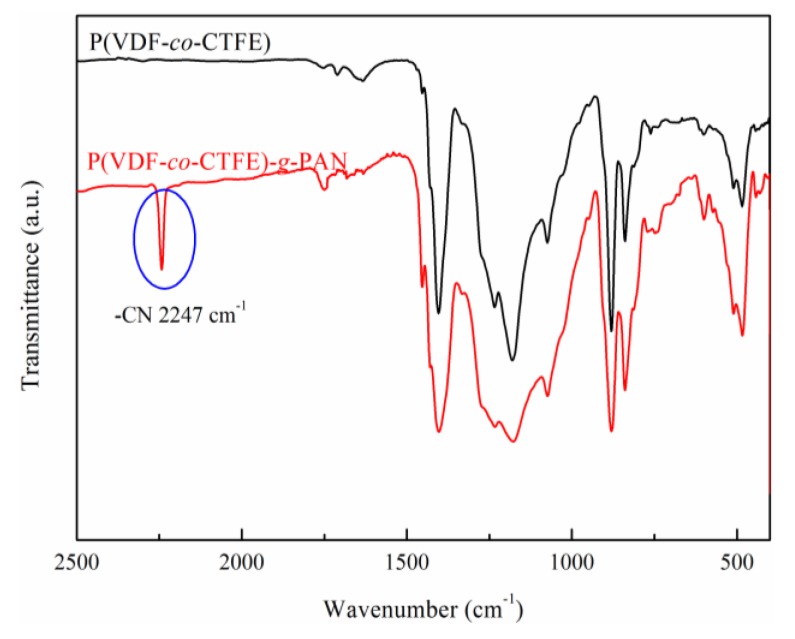
Fourier transform infrared (FTIR) spectrum of pristine P(VDF-*co*-CTFE) and P(VDF-*co*-CTFE)-*g*-PAN (Table 1, run 5).

**Figure 4:**
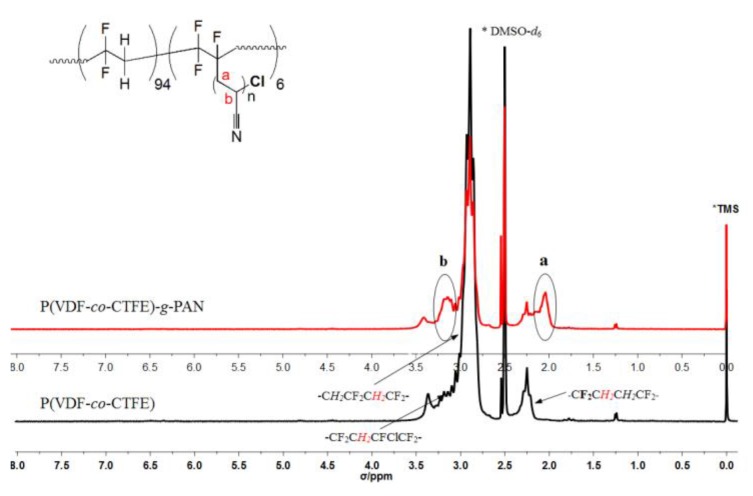
^1^H NMR of pristine P(VDF-*co*-CTFE) and P(VDF-*co*-CTFE)-*g*-PAN (Table 1, run 5).

**Figure 5:**
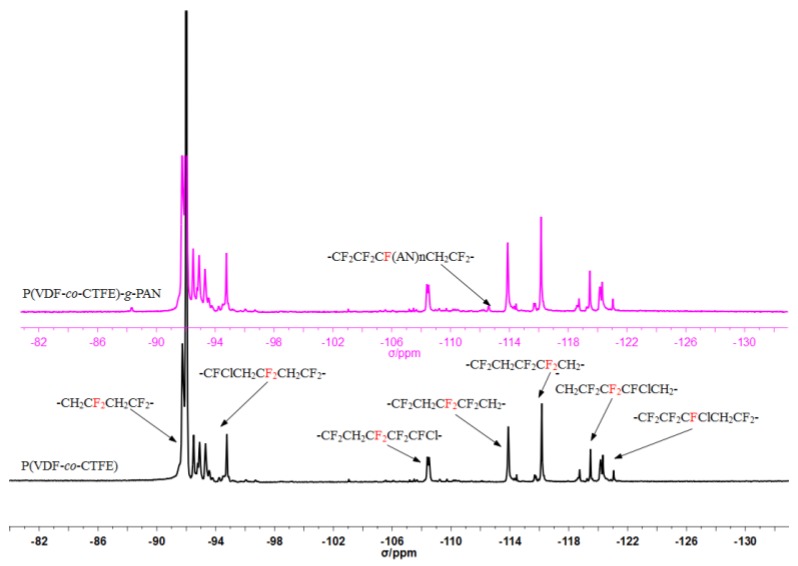
^19^F NMR of pristine P(VDF-*co*-CTFE) and P(VDF-*co*-CTFE)-*g*-PAN (Table 1, run 5).

The chemical structure of P(VDF-*co*-CTFE)-*g*-PAN was characterized by FTIR, ^1^H NMR, and ^19^F NMR. In Figure 3, the characteristic absorption peak at 2247 cm^−1^ (–CN) indicated the presence of PAN segments in the copolymer. The ^1^H NMR spectrum (Figure 4) displayed two multiple peaks of head-head and head-tail connections of VDF units (2.2–2.4 ppm and 2.7–3.2 ppm). The shoulder peak at 3.0–3.3 ppm corresponded to the proton signal of VDF adjacent to CTFE (–CF_2_CH_2_CFClCF_2_–). A new peak appearing around 1.9–2.1ppm was attributed to the proton on the methylene group of the AN unit (–CH_2_-CHCN–). Another new peak appeared at 3.0–3.3 ppm and overlapped with the proton signals of head-to-tail connections of VDF in P(VDF-*co*-CTFE), which was assigned to the methine proton (–CH_2_CHCN–). The signals of –CH_2_CHFCF_2_– from hydrogenation of CTFE unites and –CH=CFlCF_2_– from elimination of HCl from main chains were not observed. This indicated that the typical side reactions did not happen in this photoinduced Cu(II)-mediated RDRP process. The ^19^F NMR spectrum provided more information about the structure of graft copolymers. In Figure 5, the new peak appearing at 112.3 ppm was attributed to the AN units inserting into the C–Cl bond, where the C–C bond took the place of the C–Cl bond, which was consistent with the previous report [57]. No other new peaks were observed, which illustrated that the typical side reactions in traditional ATRP were avoided in this process.

**Figure 6:**
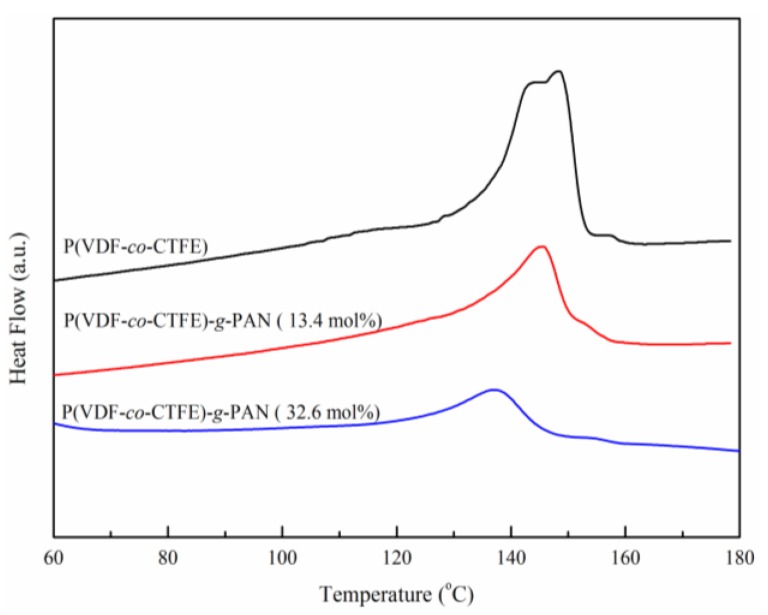
Differential scanning calorimetry (DSC) curves of pristine P(VDF-*co*-CTFE) and P(VDF-*co*-CTFE)-*g*-PAN with varied graft contents (Table 1, runs 7 and 9).

**Figure 7:**
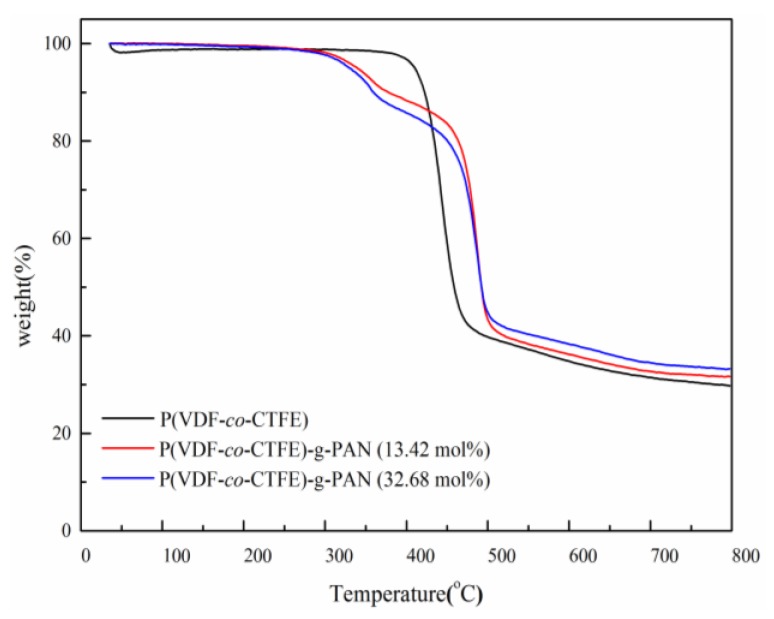
Thermogravimetric analysis (TGA) curves of pristine P(VDF-*co*-CTFE) and P(VDF-*co*-CTFE)-*g*-PAN copolymers with varied graft contents (Table 1, runs 7 and 9).

The thermal properties of P(VDF-*co*-CTFE)-*g*-PAN was explored by using DSC and TGA. In Figure 6, one endothermic peak was observed on each curve below 180 °C, which was assigned to the melting of the crystalline fluoropolymers. The melting temperature decreased from 149 to 137 °C with the increase of PAN graft content. This suggested that the crystalline degree of P(VDF-*co*-CTFE) and the crystal domain size were influenced by the introduction of PAN graft segments. The TGA (Figure 7) showed that the pristine P(VDF-*co*-CTFE) began to decompose at about 400 °C, and about 40 wt % remained at 500 °C. Meanwhile, two polymer degradation stages were observed for the P(VDF-*co*-CTFE)-*g*-PAN copolymers. The first stage started from about 300 °C, which corresponded to the decomposition of PAN segment. The second stage began from about 450 °C, which was attributed to the degradation of P(VDF-*co*-CTFE) backbone chain. It was noteworthy that the weight remaining over 500 °C was enhanced with the increase of PAN content. It was supposed that the PAN was carbonized to carbon material with higher thermal stability.

## 4. Conclusions

In summary, poly(vinylidene fluoride-*co*-chlorotrifluoroethylene)-*graft*-polyacrylonitrile (P(VDF-*co*-CTFE)-*g*-PAN) with varied graft contents were prepared via photoinduced Cu(II)-mediated reversible deactivation radical polymerization (RDRP). Upon UV irradiation, CuCl_2_/Me_6_-Tren enabled low catalyst loading and temporal control of the polymerization process. This protocol might have potential application in the area of novel dielectric materials. 

## Figures and Tables

**Table 1:** Summary of photoinduced Cu(II)-mediated RDRP of AN in DMSO with P(VDF-*co*-CTFE) as macroinitiator under UV (λ_max_ = 365 nm) irradiation for 6 h.

Run	[Cl]:[Cu]:[L]:[AN]	Conversion%	Graft content ^[a]^mol %	Graft length ^[b]^
1^[c]^	1:(1/32):(6/32):30	0	0	0
2	0:(1/32):(6/32):30	0	-	-
3	1:(0):(0):30	0	0	0
4	1:(1/32):(4/32):30	10.0	17.2	2.9
5	1:(1/32):(6/32):30	12.3	20.8	3.5
6	1:(1/32):(8/32):30	8.1	13.7	2.3
7	1:(1/64):(6/64):30	7.8	13.4	2.2
8	1:(1/32):(6/32):50	9.9	27.5	4.6
9	1:(1/32):(6/32):80	7.5	32.6	5.5

^[a]^ Graft content was calculated by ^1^H NMR according to the literature [57]. ^[b]^ Graft length equaled graft content divided by 6%. ^[c]^ Polymerization was conducted without UV.
